# Parental perceived neighborhood attributes: associations with active transport and physical activity among 10–12 year old children and the mediating role of independent mobility

**DOI:** 10.1186/1471-2458-14-631

**Published:** 2014-06-20

**Authors:** Femke De Meester, Delfien Van Dyck, Ilse De Bourdeaudhuij, Greet Cardon

**Affiliations:** 1 Department of Movement and Sport Sciences, Faculty of Medicine and Health Sciences, Ghent University, Watersportlaan 2, B-9000 Ghent, Belgium; 2 Research Foundation Flanders (FWO), Brussels, Belgium

## Abstract

**Background:**

During the last decades, the use of active travel modes declined in all age groups. Childhood is a critical time to establish lifelong healthy patterns. To develop effective interventions in this age group, insight in the correlates of health behaviors and the possible mediating factors is necessary. Among children, the role of parents may not be overlooked. Therefore, this study aimed to examine the associations of parental perceptions of neighborhood environmental attributes with active transport and total physical activity in 10–12 year old Belgian boys and girls. Furthermore, this study examined the potential mediating effect of independent mobility on these associations.

**Methods:**

In the present study, 736 10–12 year old children and their parents from 44 elementary schools in Flanders, Belgium, participated. The children were asked to wear an activity monitor and to fill in a survey questioning demographic factors and the Flemish Physical Activity Questionnaire. The parents filled in a survey concerning demographic factors, the child’s level of independent mobility and environmental perceptions (Neighborhood Environmental Walkability Scale).

**Results:**

Overall, boys reported more active transport when parents perceived more land use mix diversity, shorter distances to school, good land use mix access, higher residential density and less pleasing neighborhood aesthetics. Higher total physical activity levels were reported when parents perceived shorter distances to school and availability of walking/cycling infrastructure. None of the associations was mediated by independent mobility in boys. Girls reported more active transport when parents perceived higher residential density, more land use mix diversity, shorter distances to school, good land use mix access, available walking/cycling infrastructure and convenient recreational facilities. Girls reported higher total physical activity levels when parents perceived high residential density, good land use mix access, well-maintained and high quality walking/cycling infrastructures and more traffic safety. Independent mobility was found to be an important mediator of these associations in girls.

**Conclusions:**

Neighborhood environmental interventions to increase children’s active transport and physical activity can be effective when combined with awareness raising programs for parents. Furthermore, among girls encouraging independent mobility may contribute to behavior change.

## Background

Regular physical activity during childhood is associated with different well-known health benefits, including the maintenance of a healthy weight, protective effects on the development of diabetes mellitus type 2, promotion of bone health as well as prevention of cardiovascular disease risk factors [1-6]. Despite these health benefits, many children do not achieve the public health recommendations of 60 minutes moderate-to-vigorous physical activity per day [7].

Walking and cycling for transport, also called ‘active transport’ have been acknowledged as affordable and convenient sources of physical activity with a significant contribution to the total physical activity levels [8,9]. The results of the reviews of Davison et al. [9] and Faulkner et al. [10] both showed that children who walk or cycle to school have higher overall physical activity levels than those who use motorized transport. However, during the past decades, there has been a consistent decline in the use of active travel modes [11-15]. As childhood is a critical to establish lifelong healthy patterns, the development of effective interventions to promote physical activity and in particular active transport among children has become a public health priority in most developed countries [16].

During the last decade, ecological models have been used more frequently than the more individually-oriented models to investigate the correlates of children’s physical activity behavior [17-19]. Ecological models emphasize the importance of environmental characteristics, after the individual and social factors, in explaining physical activity behavior [20]. Interventions designed to create environments that stimulate the targeted behavior change are appealing because they have the potential for having a sustained impact on population groups rather than short-term impacts on individuals [17,20].

As a consequence of the shift from individually-oriented correlates of physical activity towards environmental-oriented correlates as opportunity to shape physical activity, empirical evidence is accumulating documenting on the role of neighborhood environmental attributes to explain children’s physical activity behavior. However, in contrast to the consistent findings in adults [21-24], the association between neighborhood environmental attributes and children’s levels of physical activity is rather blurred [25,26]. A recent review of Ding et al. (2011) concluded that in only 34 percent of the studies among children, investigating the association between perceived built environmental attributes and self-reported physical activity, a significant positive association was found. In the other 66 percent of the studies, no association could be established [25].

Within the existing evidence on the importance of neighborhood environmental attributes for active transport, more attention has been given to active transport to school than to other forms of active transport [27,28]. A recent review of D’Haese et al. (unpublished data) showed that in a number of studies high neighborhood walkability, absence of freeway crossings, high land use mix accessibility and high neighborhood safety were associated with higher levels of active transport to school in children. Nevertheless, no consistent results were found concerning the association between active transport to school and other neighborhood environmental attributes like: land use mix diversity, street connectivity, the availability of walking and cycling infrastructure, the presence of sidewalks and cycle tracks, altitude differences, aesthetics, crime and traffic safety, recreational facilities and the degree of urbanization. Furthermore, no definite conclusions could be drawn with regard to the contribution of neighborhood built environmental attributes to explain active transport during leisure time (e.g. to visit friends).

Thus, a number of reviews summarized the findings from studies on the association of neighborhood environmental attributes with physical activity or with active transport among children [25,26]. But, the findings described in these reviews are inconclusive. The inconclusiveness of these findings can possibly be attributed to the manner in which neighborhood environmental attributes were assessed, the nature of the participants and the underestimation of the accumulation of influences that affect physical activity.

As stated by Ding et al. [25] and Giles-Corti et al. [29], the inconclusiveness of reviews summarizing the findings from studies on the association of neighborhood environmental attributes with physical activity or with active transport among children is potentially influenced by the different methods to measure environmental attributes. According to Ball et al. (2008), objectively determined built environmental attributes may potentially be indirectly associated with physical activity, whereas perceptions of environmental attributes have a more direct influence on physical activity [30]. The presence of environmental attributes might therefore not automatically influence the behavior in the absence of awareness of those attributes [30].

Further, previous studies based on self-reported perceptions of neighborhood built environmental characteristics used parental perceptions as well as children’s perceptions. Parents are important gatekeepers and decision makers for their children’s physical activity and travel behavior. It may therefore be the case that parental perceptions of neighborhood environmental attributes are of greater importance than the perceptions of the children themselves.

Furthermore, as stated by Giles-Corti et al. [29] and Panter et al. [27] it is likely that the impact of the neighborhood built environmental attributes on children’s activity level is moderated or mediated by a range of factors, such as certain characteristics of the children and their parents. An important overlooked factor for the association between neighborhood environmental attributes and children’s physical activity behavior might be the independent mobility granted by the parents. Children’s independent mobility can be defined as the geographical distance from children’s home to places where they are allowed to wander when playing and socializing [31]. It is possible that the impact of the neighborhood built environmental attributes on children’s active transport and activity level may vary according to the level of independent mobility of the children. Different studies have shown that the parental perceptions of certain neighborhood environmental attributes are related to the level of independent mobility of children. Neighborhoods characterized by a shorter distance to school, less traffic danger, proximity to friends, no freeways and presence of facilities to assist active travel are found to be positively associated with the level of independent mobility of children [32-35]. This, in turn, may increase their level of active transport as different studies have shown that children who encounter less mobility restrictions have higher levels of active transport. The results of a study of Page et al., conducted in the UK, showed that 10–11 year old boys and girls with higher levels of independent mobility had higher levels of active transport [36]. Similar results were found in an Australian study of Carver et al. in the same age group [37]. Furthermore, children’s level of independent mobility was also found to be positively associated with children’s overall outdoor play [38] and accelerometer determined weekday physical activity [39].

To our knowledge no previous studies investigated the mediating role of the level of independent mobility on the associations between parental perceptions of neighborhood environmental attributes and physical activity behaviors such as active transport. When investigating these indirect pathways, it is important to take into account the gender of the children as the level of independent mobility is found to be higher in boys compared to girls [39-42].

The first aim of the present study was to examine the association of the parental perceptions of neighborhood environmental attributes with active transport and total physical activity in 10–12 year old Belgian boys and girls. Secondly, this study examined the potential mediating effect of independent mobility on the associations of the parental perceptions of neighborhood environmental attributes with the level of active transport and total physical activity of 10–12 year old boys and girls.

## Methods

### Participants and procedure

In the present study, a large sample of 10–12 year old children from 44 elementary schools in East- and West-Flanders, Belgium, participated. All children attended the last year of elementary school. To recruit the children, 148 schools were randomly selected from all elementary schools in East- and West-Flanders and the principals were contacted by phone. After this first contact, 44 principals agreed to let the final year of their school (1 class group per school) participate (response rate = 29.7%) and gave written consent. The main reasons for refusal were “no time” and “participated already in many other studies or projects”.

In total, 976 children and their parents could be reached. Both the children and their parents received an informative letter about the study with an invitation to participate. Finally, the parents of 749 children from 44 class groups agreed to let their child participate in this study and consented to be involved in the study (response rate = 76.7%).

Data collection took place between September 2010 and June 2011. During school hours and under supervision of a research assistant, the children with written consent to participate completed a questionnaire on socio-demographic variables and their own PA behavior. Furthermore, the research assistant explained the protocol of the activity monitor and its diary as the children were asked to wear an activity monitor for seven consecutive days. Every child was also given a questionnaire to be completed by one of the parents. The parental questionnaire contained questions concerning socio-demographic characteristics, neighborhood environmental perceptions and the level of independent mobility of their child. Parents completed this questionnaire at home. One week later, the research assistant visited the schools for a second time to collect the parental questionnaires, activity monitors and diaries. The parental questionnaires, activity monitors or diaries that were forgotten the second visit, were redirected by the teacher or collected by the research assistant during a third visit.

The study protocol received approval from the Ethics Committee of Ghent University Hospital.

### Measures

#### Demographic characteristics

Children’s age and gender was questioned in the child questionnaire. In the parental questionnaire, the parents were asked to fill in their own and their partner’s level of education. Educational attainment of the children’s parents was used as a proxy measure of children’s SES. The educational level of the child’s mother and father was determined based on four options: less than high school, completed high school, completed college or completed university. The educational level of mother and father was coded into ‘reached a college or a university level’ or ‘did not reach a college or a university education level’.

#### Neighborhood built environmental attributes

To measure the parental perceptions of neighborhood built environmental attributes, the parent version of the Neighborhood Environmental Walkability Scale for Youth (NEWS-Y) was used [43]. The NEWS-Y has acceptable to good test-retest reliability with an intra-class correlation coefficient between 0.56 and 0.87 [44] for parents of children between 5 and 10 years. The neighborhood built environmental attributes questioned were residential density, land use mix diversity, distance to school, land use mix access, street network connectivity, availability of walking and cycling infrastructure, maintenance and quality of walking and cycling infrastructures, aesthetics of the neighborhood, convenience of recreational facilities and crime and traffic safety. The neighborhood was defined as the immediate environment around the house within a distance of 1 kilometer (10 – 15 minutes walking distance). The NEWS-Y scoring guidelines were used to calculate the subscales. Following the NEWS-Y scoring guidelines [45], the subscale residential density was computed by the following formula: score on question 1a (single family residences) + 12*score on question 1b (row houses) + 25*score on question 1c (apartments). The other subscales were calculated by taking the mean of the different item scores. The content, response options and descriptive statistics are given in Table 1.

**Table 1:** Content, response options and descriptive statistics of the parental perceived neighborhood built environmental attributes

	**Content of the item**	**Response category**	**Mean score boys**	**Mean score girls**
Residential density (3 items)	Presence of different types of residences (e.g. detached single family residences, row houses, apartments)	5-point scale^a^	78.8 (26.9)	79.5 (26.3)
Land use mix diversity (9 items)	Distance to local facilities (e.g. supermarket, post office, library)	5-point scale^b^	3.4 (0.9)	3.4 (0.9)
Land use mix access (5 items)	Access to neighborhood services for their child (e.g. ease to walk to public transport, ease to walk to school)	5-point scale^c^	3.6 (1.1)	3.6 (1.0)
Distance to school (1item)	Distance to the school of the adolescent	5-point scale^b^	2.9 (1.4)	3.0 (1.4)
Connectivity (3 items)	Connectedness of street network (e.g. presence of intersections, dead-end streets, alternate routes)	5-point scale^c^	3.3 (0.7)	3.3 (0.7)
Availability of walking and cycling infrastructure (4 items)	Availability of walking and cycling infrastructure (e.g. footpaths and cycling lanes in most streets, footpaths and cycling lanes separated from streets )	5-point scale^c^	2.7 (0.9)	2.8 (0.9)
Quality and maintenance of walking and cycling infrastructure (5 items)	Quality and maintenance of walking and cycling infrastructure (e.g. maintenance of cycling lanes and footpaths, presence of lighting)	5-point scale^c^	3.1 (1.0)	3.1 (0.9)
Aesthetics (4 items)	Presence of aesthetic features (e.g. green spaces, attractive buildings, streets free from litter and graffiti)	5-point scale^c^	3.6 (0.8)	3.5 (0.7)
Safety for traffic (6 items)	Perceived safety from traffic problems (e.g. speed of traffic in neighborhood, availability of pedestrian crossings and traffic signals)	5-point scale^c^	2.9 (0.7)	2.8 (0.7)
Safety for crime (4 items)	Perceived safety from crime (e.g. crime prevalence in the neighborhood, perceived safety from strangers)	5-point scale^c^	3.5 (0.8)	3.4 (0.7)
Convenience of recreation facilities (5 items)	Distance to PA facilities (e.g. sports field, sports hall, swimming pool, park)	5-point scale^c^	3.5 (0.9)	3.4 (0.9)

PA: physical activity.

^a^none, a few, about half, a lot, all.

^b^> 30 min, 21–30 min, 11–20 min, 6–10 min, 1–5 min.

^c^strongly disagree, somewhat disagree, neither agree or disagree, somewhat agree, strongly agree.

Note: all perceived built environmental attributes were positively scored: higher score = more walkable.

#### Independent mobility

To determine children’s level of independent mobility, two items were included in the parent questionnaire “How far is your child allowed to cycle from home without adult accompaniment?” and “How far is your child allowed to walk from home without adult accompaniment?”. Response categories were: not, 0 m-200 m, 200 m-500 m, 500 m-1 km, 1 km-3 km, 3 km-5 km, 5 km-10 km, +10 km. The question “How far is your child allowed to cycle from home without adult accompaniment?” was found to have good reliability with an ICC of 0.79 [46]. The second question was informed by this question. The highest score on these two items was taken as an indication of how far they were allowed to walk or cycle from home without adult accompaniment.

#### Physical activity

The Flemish Physical Activity Questionnaire (FPAQ) was used to determine the duration (hours and minutes per day) of school related active transportation (walking and cycling to and from school), walking and cycling for transport during leisure time and total physical activity level. The total physical activity level [47] was calculated using the minutes of all activities questioned in the FPAQ (walking and cycling to and from school, walking and cycling for transport during leisure time, school-related sporting activities and leisure time sporting activities). The FPAQ was found to be a reliable and reasonably valid questionnaire for the assessment of these different dimensions of physical activity in youth [47].

To measure free-living step counts, the children were asked to wear an activity monitor for 7 consecutive days, including two weekend days. The Yamax Digiwalker SW-200 (Yamax cooperation, Tokio, Japan) and the Actigraph accelerometer, model GT1M (Actigraph MTI, Manufacturing Technology Inc., Pensacola, FL, USA) were used. The Yamax Digiwalker has been acknowledged as a valid, accurate and reliable instrument to measure free-living step-counts [48]. The GT1M accelerometer has demonstrated good reliability for measuring steps [49]. Evidence exists that neither accelerometers nor pedometers are affected by reactivity among adolescents [50,51]. Although the step counts measured by the Yamax Digi-walker CW-701 (the update of the Yamax Digiwalker SW-200) have been shown to be highly correlated with the step counts of the GT1M accelerometer (r = 0.78), the overall agreement between the step counts of both monitors is rather low [52]. In the study of Kinnunen et al. (2011), the 95% limits of agreement ranged between −2690 to 2656 steps/day for the mean value (mean of accelerometer and pedometer steps/day = 6026). Further, the limits of agreement varied substantially over the range of values. At the lowest recorded step count (mean of accelerometer and pedometer steps/day = 906) the accelerometer was on average recording more steps/day than the pedometer. In contrast, at the highest step count value (mean of accelerometer and pedometer steps/day = 12,018) the accelerometer recorded less steps/day than the pedometer on average [52]. To overcome this problem, all analyses were controlled for the type of monitor used.

The children were asked to wear the activity monitor (pedometer or accelerometer) during waking hours but to remove the monitor for aquatic activities and for activities that prohibit activity monitors. Together with their activity monitor, all children received a diary. Children who wore a pedometer were asked to record the dates, steps taken at the end of the day and a description and duration of the activities for which the pedometer was removed in the diary. The children who wore an accelerometer were asked to record the duration and a description of the activities for which the accelerometer was removed. The children who wore an accelerometer were not asked to record the steps taken at the end of the day because accelerometers have a memory and are able to save the steps taken during the entire recording period. At the end of the measurement period, the activity monitors were collected and the accelerometer determined step count data were downloaded (Actilife software version 4.1.0). For every minute of reported moderate or vigorous physical activity for which the activity monitor was removed, 150 steps were added to the daily number of reported step counts [53]. Among children, the importance of including non-wear activities registered in diaries when using activity monitors has been demonstrated previously [54].

### Data analyses

Preliminary analyses were conducted to obtain descriptive information about the demographic characteristics of the study sample and the study variables (children’s level of independent mobility, self-reported minutes per day of active transport, total physical activity level and objectively (pedometer or accelerometer) determined daily number of steps) using SPSS 17.0.

Tests for normal distribution revealed some skewed physical activity variables (self-reported school related active transportation, walking and cycling for transport during leisure time, total physical activity level). To obtain distributions that more closely approximated symmetry, logarithmic transformations of these variables were conducted, and the transformed variables were used in the analyses [55]. For ease of interpretation, summary data of untransformed physical activity variables are reported in minutes/day (Table 2).

**Table 2:** Demographic characteristics and descriptive statistics for adolescents’ level of independent mobility and physical activity behavior

	**Total sample**	**Girls**	**Boys**
**Age: mean (SD)**	11.2 (0.5)	11.1 (0.5)	11.2 (0.6)
**Weight status: %**			
Overweight	12.0	14.2	10.0
Obese	5.0	5.1	5.0
**Gender: %**			
Male	51.9		
Female	48.1		
**Educational level: % Mother:**			
No college/university degree	45.6	48.2	43.2
College or university degree	54.4	51.8	56.8
**Father**			
No college/university degree	54.2	55.1	53.4
College or university degree	45.8	44.9	46.6
**Self-reported physical activity (min/day (SD))**			
Active transport to and from school	10.2 (12.7)	9.3 (11.8)	11.2 (13.5)
Walking for transport during leisure time	9.0 (12.4)	7.8 (11.2)	10.2 (13.3)
Cycling for transport during leisure time	10.5 (13.1)	8.1 (11.2)	12.8 (14.3)
Total physical activity	81.3 (43.1)	68.7 (35.4)	93.2 (46.3)
**Daily step counts (steps/day (SD))**	10 766 (3503)	9739 (2979)	11 740 (3684)
**Level of independent mobility (%)**			
Not	3.5	5.7	1.4
0 m – 200 m	1.3	1.8	0.8
200 m – 500 m	8.1	7.5	8.7
500 m – 1 km	17.7	20.9	14.8
1 km – 3 km	32.6	31.6	33.5
3 km – 5 km	22.4	20.9	23.7
5 km – 10 km	10.0	7.8	12.0
+10 km	4.5	3.9	5.0

**Figure 1:**
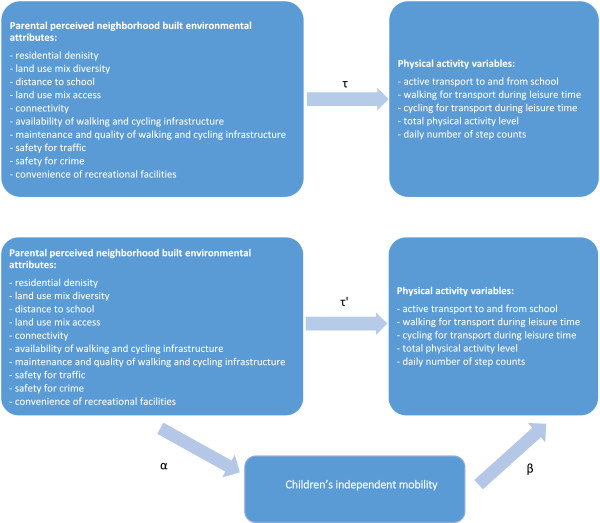
Path diagram for the mediational effect of children’s independent mobility on the association of neighborhood environmental perceptions with the children’s level of physical activity.

Furthermore, all analyses were done separately for boys and girls given the existing evidence that boys’ level of independent mobility is higher than girls’ level of independent mobility [36,39-42].To examine the associations between the parental perceptions of neighborhood environmental attributes and self-reported minutes/day of active transport to and from school, walking for transport during leisure time, cycling for transport during leisure time, total physical activity level and objectively determined number of steps/day, multiple linear regression analyses were conducted using MLwin version 2.22. The dependent variables were self-reported minutes/day of active transport to and from school, walking for transport during leisure time, cycling for transport during leisure time, total physical activity level and objectively determined number of steps/day. These linear regression analyses provided τ-coefficients (Figure 1).

To investigate if children’s level of independent mobility mediated the association of neighborhood environmental perceptions with their level of physical activity, the product-of-coefficient test of McKinnon et al. was used (Figure 1) [56]. This test included three steps that were conducted in MLwin version 2.22. In a first step, the effects of the neighborhood environmental perceptions on the potential mediator (children’s independent mobility) were examined by regressing children’s independent mobility onto the neighborhood environmental perceptions. Step one was conducted only for the parental perceptions that showed a significant direct association with the children’s level of active transport, total physical activity level and objectively determined daily number of step counts. This step provided estimates of the α-coefficients.

In the second step, the independent effect of the potential mediator on the dependent physical activity variables was investigated by regressing the dependent physical activity variables onto the neighborhood environmental perceptions and the potential mediator (children’s level of mobility) (ß-coefficients). Step two was only conducted for the parental perceptions that showed a significant association with the potential mediator.

In the third step, the mediated effect was calculated by multiplying the two coefficients (αß).

The statistical significance of the mediated effect was calculated by dividing the product-of-coefficients (αß) by its standard error. Moreover, the proportion mediated was calculated by dividing the product-of-coefficient (αß) by the total main effect of the neighborhood environmental perceptions on the dependent physical activity variables (τ).

For all analyses, 95% confidence intervals (CI) were reported. All analyses were controlled for two proxy measures of individual SES (educational attainment of mother and father). Clustering of individuals in schools was taken into account by using multi-level modeling with children at the first level and schools at the second level. All analyses including the daily step counts as dependent variable were controlled for the type of monitor (pedometer or accelerometer).

## Results

### Sample characteristics

Table 2 provides an overview of the descriptive statistics for demographic characteristics, physical activity behavior and independent mobility for the total sample and by gender. In total, 736 (98.3%) children completed the questionnaire and handed it back to the research assistant. From 93.5% of the parents (n = 701), a complete questionnaire returned to school. In 92.1% the children and the parent, who filled in the questionnaire, had the same address. In 6.3% the children lived partly with the parent who filled in the questionnaire. In 1.6% the child did not live with the person who filled in the questionnaire.

In total, 649 children (86.6%) had complete step count data, 370 children wore a pedometer and 279 an accelerometer. The sample consisted of 354 girls (48.1%) and 382 boys (51.9%). Mean age was 11.2 ± 0.5 years. The children reported on average 10.2 (±12.7) minutes/day of walking and cycling to and from school, 9.0 (±12.4) minutes/day of walking during leisure time, 10.5 (±13.1) minutes/day of cycling during leisure time and 81.3 (±43.1) minutes/day of physical activity. On average, the children took 10 766 (±3503) steps/day.

Most of the boys and girls were allowed to go between 500 m and 5 km from home unsupervised. A significant difference in independent mobility was found between boys and girls (t = 3.438; p < 0.01). Boys were allowed to go further from home unsupervised using active transport compared to girls.

### Main effects of neighborhood environmental perceptions on active transport, the total physical activity level and the daily amount of step counts (τ-coefficients)

Table 3 shows the main effects of neighborhood environmental perceptions on active transport, the total physical activity level and the daily amount of step counts among boys and girls.

**Table 3:** Main effects of neighborhood environmental perceptions on physical activity variables among boys and girls (τ-coefficients)

	***Active transport to and from school***		***Walking for transport during leisure time leisure***		***Cycling for transport during leisure time leisure***		***Overall level of physical activity***		***Daily number of step counts***	
**MAIN EFFECTS AMONG BOYS**	**τ (SE)**	**95% CI**	**τ (SE)**	**95% CI**	**τ (SE)**	**95% CI**	**τ (SE)**	**95% CI**	**τ (SE)**	**95% CI**
**Residential density**	0.001 (0.001)	−0.001-0.003	**0.004 (0.001)**	**0.002-0.006**	0.002 (0.001)	0.000-0.004	0.000 (0.001)	−0.002-0.002	7.322 (8.766)	−9.859-24.503
**Land use mix diversity**	**0.108 (0.036)**	**0.037-0.179**	**0.076 (0.039)**	**0.000-0.152**	0.050 (0.040)	−0.028-0.128	0.005 (0.015)	−0.024-0.034	−4.615 (226.984)	−449.504-440.274
**Distance to school**	**0.129 (0.023)**	**0.084-0.174**	**0.065 (0.025)**	**0.016-0.114**	**0.062 (0.026)**	**0.011-0.113**	**0.026 (0.010)**	**0.006-0.046**	−23.777 (149.181)	−316.172-268.618
**Land use mix access**	**0.185 (0.030)**	**0.126-0.244**	0.061 (0.033)	−0.004-0.126	0.036 (0.034)	−0.031-0.103	0.022 (0.013)	−0.003-0.047	147.627 (195.926)	−236.388-531.642
**Connectivity**	0.091 (0.049)	−0.005-0.187	0.015 (0.052)	−0.087-0.117	0.010 (0.053)	−0..094-0.114	0.012 (0.020)	−0.027-0.051	423.100 (312.254)	−206.558-1052.758
**Availability of walking and cycling infrastructure**	0.070 (0.037)	−0.003-0.143	−0.020 (0.039)	−0.096-0.056	0.047 (0.040)	−0.031-0.125	**0.033 (0.015)**	**0.004-0.062**	72.449 (233.694)	−385.591-530.489
**Maintenance and quality of walking and cycling infrastructure**	0.035 (0.033)	−0.030-0.100	−0.021 (0.034)	−0.088-0.046	0.032 (0.035)	−0.037-0.101	0.015 (0.013)	−0.010-0.040	36.869 (202.103)	−359.253-432.991
**Aesthetics**	−0.064 (0.047)	−0.156-0.028	**−0.116 (0.048)**	**−0.210-(−0.022)**	−0.049 (0.050)	−0.147-0.049	−0.012 (0.019)	−0.049-0.025	−214.570 (292.276)	−787.431-358.291
**Safety for traffic**	0.055 (0.051)	−0.045-0.155	0.029 (0.054)	−0.077-0.135	0.051 (0.056)	−0.059-0.161	−0.010 (0.021)	−0.051-0.031	−11.255 (316.694)	−631.975-609.465
**Safety for crime**	−0.010 (0.046)	−0.100-0.080	−0.076 (0.047)	−0.168-0.016	−0.028 (0.049)	−0.124-0.068	−0.012 (0.019)	−0.049-0.025	−205.080 (287.894)	769.352-359.192
**Convenience of recreational facilities**	0.050 (0.040)	−0.028-0.128	−0.011 (0.042)	−0.093-0.071	−0.022 (0.043)	−0.106-0.062	−0.006 (0.017)	−0.039-0.027	150.952 (250.034)	−339.115-641.019
**MAIN EFFECTS AMONG GIRLS**	**τ (SE)**	**95% CI**	**τ (SE)**	**95% CI**	**τ (SE)**	**95% CI**	**τ (SE)**	**95% CI**	**τ (SE)**	**95% CI**
**Residential density**	**0.004 (0.001)**	**0.002-0.006**	**0.005 (0.001)**	**0.003-0.007**	0.001 (0.001)	−0.001-0.003	**0.001 (0.000)**	**0.001-0.001**	6.298 (7.407)	−8.220-20.816
**Land use mix diversity**	**0.089 (0.037)**	**0.016-0.162**	**0.102 (0.039)**	**0.026-0.178**	−0.001 (0.037)	−0.074-0.072	0.026 (0.015)	−0.003-0.055	21.472 (181.128)	−333.539-376.483
**Distance to school**	**0.126 (0.024)**	**0.079-0.173**	0.046 (0.026)	−0.005-0.097	−0.007 (0.025)	−0.056-0.042	0.015 (0.010)	−0.005-0.035	−84.890 (122.967)	−325.905-156.125
**Land use mix access**	**0.143 (0.032)**	**0.080-0.206**	**0.129 (0.035)**	**0.060-0.198**	−0.015 (0.034)	−0.082-0.052	**0.035 (0.014)**	**0.008-0.062**	286.596 (167.068)	−40.857-614.049
**Connectivity**	0.008 (0.050)	−0.090-0.106	0.019 (0.054)	−0.087-0.125	0.013 (0.051)	−0.087-0.113	−0.024 (0.021)	−0.065-0.017	182.826 (248.738)	−304.700-670.352
**Availability of walking and cycling infrastructure**	**0.101 (0.039)**	**0.025-0.177**	**0.086 (0.042)**	**0.004-0.168**	−0.000 (0.040)	−0.079-0.078	0.004 (0.017)	−0.029-0.037	−143.455 (196.440)	−529.477-241.567
**Maintenance and quality of walking and cycling infrastructure**	0.025 (0.037)	−0.048-0.098	0.050 (0.039)	−0.026-0.126	0.020 (0.037)	−0.053-0.093	**0.042 (0.015)**	**0.013-0.071**	110.504 (185.024)	−252.143-473.151
**Aesthetics**	−0.089 (0.050)	−0.187-0.009	−0.077 (0.053)	−0.181-0.027	−0.043(0.050)	−0.141-0.055	0.012 (0.021)	−0.029-0.053	−67.354 (250.140)	−557.628-422.920
**Safety for traffic**	−0.002 (0.050)	−0.100-0.096	0.078 (0.054)	−0.028-0.184	0.060 (0.051)	−0.040-0.160	**0.053 (0.021)**	**0.012-0.094**	139.887 (253.962)	−357.879-637.653
**Safety for crime**	0.039 (0.052)	−0.063-0.141	−0.094 (0.055)	−0.202-0.014	0.041 (0.052)	−0.061-0.143	0.023 (0.022)	−0.020-0.066	−44.984 (257.382)	−549.453-459.485
**Convenience of recreational facilities**	0.059 (0.036)	−0.012-0.130	**0.108 (0.039)**	**0.032-0.184**	−0.028 (0.037)	−0.101-0.045	0.022 (0.015)	−0.007-0.051	25.069 (184.481)	−336.514-386.652

Note: All regression models were adjusted for educational attainment of mother and father and clustering by schools. All regression models including the daily step counts as dependent variable were controlled for type of monitor (pedometer or accelerometer). The significant effects were shown in bold.

The results of the regression analyses revealed that boys reported more minutes of *active transport to and from school* when their parents perceived more land use mix diversity, a shorter distance to the school of their child and good land use mix access.

When parents perceived a higher residential density, more land use mix diversity, a shorter distance to school and less pleasing neighborhood aesthetics, their male children reported more *walking for transport during leisure time*. Furthermore, the parental perception of a shorter distance to school was associated with more *cycling for transport during leisure time* and the parental perception of a shorter distance to school and availability of walking and cycling infrastructure were associated with more self-reported *total physical activity*. No associations were found between the parental neighborhood environmental perceptions and the *objectively determined daily number of step counts*.

Among girls (Table 3), the regression analyses revealed that when parents perceived a higher neighborhood residential density, more land use mix diversity, a shorter distance to the school of their child, good land use mix access and availability of walking and cycling infrastructure, their female children reported more minutes of *active transport to and from school.*

More *walking for transport during leisure time* was associated with a higher parental perception of residential density, more land use mix diversity, good land use mix access, availability of walking and cycling infrastructure and convenient recreational facilities. When parents perceived high neighborhood residential density, good land use mix access, well-maintained and high-quality walking and cycling infrastructures and more safety for traffic, their children reached a higher level of *total physical activity*. For the number of minutes of *cycling for transport during leisure time* and the *objectively determined daily number of step counts* no associations with the parental perceptions of neighborhood environmental attributes were found.

#### Step 1: effects of the neighborhood environmental perceptions on the potential mediator (α coefficients)

Girls had higher levels of independent mobility when their parents perceived a higher neighborhood residential density, good land use mix access, high availability of walking and cycling infrastructure, more safety for traffic and convenient recreational facilities. Among boys, no significant association was found of the parental perceptions of residential density, land use mix diversity, distance to school, land use mix access, availability of walking and cycling infrastructures and aesthetics with their level of independent mobility (Table 4).

**Table 4:** Regression analyses for the possible mediated effects of independent mobility among boys and girls

**BOYS: potential mediator: independent mobility**	**α (SE)**	**95% CI**	**B (SE)**	**95% CI**	**αß (SE)**	**95% CI**	**Proportion Mediated (%)**
**Residential density**	−0.002 (0.003)	−0.008 – 0.004					
**Land use mix diversity**	−0.128 (0.081)	−0.287 – 0.031					
**Distance to school**	−0.045 (0.053)	−0.149 – 0.059					
**Land use mix access**	0.076 (0.071)	−0.063 – 0.215					
**Availability of walking and cycling infrastructure**	−0.083 (0.083)	−0.246 – 0.080					
**Aesthetics**	0.078 (0.104)	−0.126 – 0.282					
**GIRLS: potential mediator: independent mobility**	**α (SE)**	**95% CI**	**B (SE)**	**95% CI**	**αß (SE)**	**95% CI**	**Proportion Mediated (%)**
**Residential density**	**0.008(0.003)**	**0.002 – 0.014**					
*Active transport to and from school*			**0.059(0.024)**	**0.012 – 0.106**	**0.000 (0.000)**	**0.000 – 0.001**	**11.8%**
Walking for transport during leisure time leisure			0.038 (0.026)	−0.013 – 0.089			
*Total physical activity level*			**0.054 (0.010)**	**0.034 – 0.074**	**0.000 (0.000)**	**0.000 – 0.001**	**43.2%**
**Land use mix diversity**	0.050(0.092)	−0.130 – 0.230					
**Distance to school**	−0.011(0.061)	−0.131 -0.109					
**Land use mix access**	**0.250(0.080)**	**0.093 – 0.407**					
*Active transport to and from school*			**0.047(0.022)**	**0.004 – 0.090**	0.012 (0.007)	−0.001 – 0.0025	
*Walking for transport during leisure time leisure*			0.028 (0.025)	−0.021 – 0.077			
*Total physical activity level*			**0.050 (0.009)**	**0.032 – 0.068**	**0.013 (0.005)**	**0.004 – 0.023**	**37.7%**
**Availability of walking and cycling infrastructure**	**0.200(0.099)**	**0.006 – 0.394**					
*Active transport to and from school*			**0.057(0.023)**	**0.012 – 0.102**	0.011 (0.007)	−0.003 – 0.026	
*Walking for transport during leisure time leisure*			0.035 (0.025)	−0.014 – 0.084			
**Maintenance and quality of walking and cycling infrastructure**	0.108(0.091)	−0.070 – 0.286					
**Safety for traffic**	**0.411(0.123)**	**0.170 – 0.652**					
*Total physical activity level*			**0.051 (0.009)**	**0.033 – 0.069**	**0.021 (0.007)**	**0.007 – 0.035**	**39.5%**
**Convenience of recreational facilities**	**0.205(0.091)**	**0.027 – 0.383**					
*Walking for transport during leisure time leisure*			0.037 (0.025)	−0.012 – 0.085			

SE, standard error; CI confidence interval.

Notes: α-coefficients were estimated by regressing children’s level of independent mobility onto the neighbourhood environmental perceptions.

ß-coefficients were estimated by regressing the dependent active transport and physical activity variables onto the neighbourhood environmental perceptions and children’s level of independent mobility.

αß-coefficients represent the mediated effect.

All regression models were adjusted for educational attainment of mother and father and clustering by schools.

All regression models including the daily step counts as dependent variable were controlled for type of monitor (pedometer or accelerometer).

The significant effects were shown in bold.

#### Step 2: effect of the potential mediator on the dependent physical activity variables (ß coefficients)

Among girls, the level of independent mobility showed a positive association with the self-reported minutes of active transport to and from school and total physical activity after controlling for the parental perception of residential density. Similar results were found after adjustment for the parental perception of land use mix access.

After adjustment for the parental perception of the availability of walking and cycling infrastructure, the level of independent mobility was positively associated with the self-reported number of active transport to and from school. Furthermore, after adjustment for the parental perception of safety for traffic in the neighborhood, the level of independent mobility was positively associated with the total physical activity level.

No association was found between the level of independent mobility and the self-reported number of minutes of walking for transport during leisure time after taking into account the parental perceptions of the neighborhood residential density, land use mix access, the availability of walking and cycling infrastructure and the convenience of recreational facilities (Table 4).

### Mediated effects of independent mobility on the associations between neighborhood environmental perceptions and PA (αß-coefficients)

Among girls, the level of independent mobility significantly mediated the association of the parental perception of residential density with active transport to and from school (11.8%) and the total level of PA (43.2%).

Furthermore, the level of independent mobility mediated 37.7% of the association between the parental perception of land use mix access and total PA.

Finally, 39.5% of the association between the parental perception of safety for traffic and the self-reported total level PA was mediated by the level of independent mobility (Table 4).

The level of independent mobility was no significant mediator of the association between the parental perception of land use mix access and active transport to and from school. The level of independent mobility was also no significant mediator of the association between the parental perception of the availability of walking and cycling infrastructure and active transport to and from school.

## Discussion

The first aim of this study was to examine the association of the parental perception of neighborhood environmental attributes with active transport and the total physical activity level among 10–12 year old Belgian boys and girls. The results presented in this paper clearly indicate that among 10–12 year old Belgian boys and girls, the way in which their parents perceive their neighborhood is related to their level of active transport and to a lesser extent to their total physical activity level.

Specifically, a high degree of residential density, a short distance to school, a high degree of land use mix diversity, accessible neighborhood services and available walking and cycling infrastructures were identified as important parental perceptions to explain physical activity in boys and girls of this age group. The existing reviews summarizing the results of studies examining the association between neighborhood environmental attributes and physical activity behavior among children repeatedly concluded that for a lot of neighborhood built environmental attributes no definite conclusions could be drawn with regard to their contribution to explain active transport and overall physical activity level [25,26]. However, in these reviews no distinction was made between child and parental perceptions of neighborhood environmental attributes to draw conclusions. Probably, parental perceptions of environmental attributes are more important to explain active transportation and total physical activity than perceptions of the children themselves. This is also confirmed in a previous Belgian study, investigating the association between children’s perception of neighborhood environmental attributes and active transport among 13–15 year olds [57]. The results of this previous study showed that some perceived environmental attributes were found to be important for active transport to school, but the importance of the perceived environmental attributes was rather negligible for active transport during leisure time. Although the age group of that study was not comparable with the present study age group, it appears that parental perceptions of environmental attributes might be of greater importance than the perceptions of the children themselves, certainly to explain active transport during leisure time. Additional research is certainly needed to confirm this conclusion. However, if confirmed, this finding emphasizes the need to incorporate environmental changes but also awareness raising programs directed to parents to substantially increase active transport and physical activity in children.

Thus, the results of this study showed that in 10–12 year old boys and girls the parental perceptions of different neighborhood environmental attributes are associated with different forms of active transport and in some cases with their self-reported total physical activity level. The significant associations found with the self-reported total physical activity level can mostly be explained by the associations found with the active transport variables as the total physical activity level is a sum of different types of physical activity including the active transport variables. However, for some neighborhood environmental attributes a significant association was found with the total physical activity level while no significant association was found with the different active transport behaviors (i.e. the parental perceptions of the availability of walking and cycling infrastructure among boys and the parental perceptions of safety for traffic and the maintenance/quality of walking and cycling infrastructure among girls). However, it is possible that some small, non-significant correlations between the environmental characteristics and the different active transport behaviors exist and accumulate which may give a significant association with the total physical activity level.

Furthermore, no associations were found between the parental perceptions of neighborhood environmental attributes and the objectively determined daily step counts. The activity monitors used to determine the daily step counts are able to measure ambulatory movement; however, they sometimes do not detect or underestimate some types of physical activity (e.g. bicycling). Furthermore, activity monitors are often removed to undertake certain activities (e.g. swimming, marterial arts). We used non-wearing diaries to register the activities performed without an activity monitor and adjusted the daily amount of step counts based on the reported number of minutes of non-wear activities. However, the children were not asked to register cycling activities in the non-wearing diaries. Consequently, the registration of cycling activities for which the activity monitor was not removed may be inaccurate. As cycling is a common form of active transport among Belgian children [58] this can be a possible explanation for the lack of associations with the objectively determined daily step counts.

The second aim of this study was to investigate the possible mediating role of the level of independent mobility on the association of the parental perception of neighborhood environmental attributes with the level of active transport and the total physical activity level. The data of this study showed that about 50% of the children in this age group were allowed to walk or cycle between 1 km and 5 km from home without parental accompaniment. The studies found in the literature, investigating the level of independent mobility of children, operationalized the term independent mobility in different ways. To our knowledge no recent other study operationalized the level of independent mobility in terms of the geographical distance from youth’s home to places where they are allowed to wander when playing and socializing [31]. This makes comparison difficult. Consistent with other studies [37,39-41], the results of this study showed a marked difference in the level of independent mobility between boys and girls. Boys were allowed to walk or cycle further from home without supervision than girls of the same age. Parents are clearly more protective of daughters [37].

The association between the parental perceptions of neighborhood environmental attributes and the level of independent mobility was different between girls and boys. The level of independent mobility of female children was higher in neighborhoods that were perceived as more activity-friendly or walkable by the parents of the children. A higher degree of residential density in the neighborhood, higher land use mix accessibility, high availability of walking and cycling infrastructure, more safety for traffic and convenient recreational facilities were associated with higher levels of independent mobility among girls. Among boys, none of the parental perceptions of neighborhood environmental attributes that were found to be associated with active transport or the total physical activity level, showed a significant association with the level of independent mobility. Thus, out of these results we can conclude that among boys the level of independent mobility is not dependent of how parents perceive their direct neighborhood environment while among girls, the more activity friendly or ‘walkable’ the parents perceive their neighborhood, the less restricted girls are. Comparable studies relating neighborhood environmental attributes to the level of independent mobility also showed the significance of the neighborhood environment for the willingness of parents to allow their children to be active independently [33,35,39,40,59]. These studies also found differences between boys and girls. However, in none of these studies the distinctness was so obvious.

The environmental attributes questioned, are the environmental attributes of the direct neighborhood environment. The parents reported their perception about the neighborhood environment within walking distance (within a 10–15 minute walk from their homes). Since the level of independent mobility for a lot of adolescents (and especially for boys) is higher, it is possible that the environmental attributes at a further distance than the attributes within the direct neighborhood will contribute to explaining the level of independent mobility. Future research should look further into this.

Higher levels of independent mobility were related to higher levels of active transport to school and higher total physical activity levels, but no association was found with the daily amount of step counts. These findings concur with the conclusions drawn in the existing international literature. Page et al. (2009) and also Carver et al. (2010) investigated the relation between the level of independent mobility and the level of physical activity among 10–11 year old children. In both studies, independent mobility appeared to be an important correlate of the level of active transport and physical activity for this age group [37]. Since active commuting has been acknowledged by a number of studies as a physical activity behavior with a significant contribution to the total physical activity level, this has important implications for health promotion. Parents may limit their child’s level of independent mobility and consequently restrict their child’s opportunities to be physically active. Encouraging independent mobility may therefore contribute to increasing levels of active transport and as a consequence the total physical activity level.

The mediating analyses showed that in boys none of the associations between the parental perceptions of neighborhood environmental attributes and active transport or total physical activity was mediated by independent mobility. Among girls on the other hand, the level of independent mobility mediated the association of the parental perception of residential density with active transport to and from school (11.8%) and with the total self-reported level of physical activity (43.2%). Furthermore, the associations between the parental perception of land use mix access and the parental perception of safety for traffic with the total physical activity level were mediated respectively for 37.7% and 39.5% by the level of independent mobility. Based on these results, we might assume that independent mobility is of higher importance in girls than in boys. Promoting the level of physical activity in girls, through improved neighborhood environmental attributes, may be more effective if the mobility restrictions for girls are targeted as well. However, to our knowledge, this is the first time that the possible mediating role of the level of independent mobility in the association of the neighborhood environmental attributes with children’s level physical activity has been investigated. So, results cannot be compared and these results should first be confirmed in other studies before drawing definite conclusions.

Strengths of this study included the use of both objective and self-reported measures to assess physical activity. Furthermore, different physical activity behaviors related to distinct physical activity contexts were included. This allowed us to investigate the importance of the parental perceptions of neighborhood environmental attributes for specific physical activity behaviors in specific contexts. This is important for the design of future physical activity interventions. Furthermore, this study included a relatively large study sample.

Limitations of the present study included the cross-sectional study design, which does not permit to infer a causal relationship. Secondly, adolescents were not asked to register cycling activities for which the activity monitor was not removed. As activity monitors are not able to register cycling behavior accurately, this has a reflection on the daily amount of step counts. Third, step-counts were determined using the Yamax Digi-Walker CW701 and the GT1M accelerometer. Although the step counts measured by the Yamax Digi-walker CW-701 have been shown to be highly correlated with the step counts of the GT1M accelerometer, the overall agreement between the step counts of both monitors is rather low [52]. To overcome this problem, all analyses were controlled for the type of monitor used. Finally, the level of independent mobility was determined using self-reported data. The use of objective measures of GIS- and GPS-technologies in combination with self-reported data would ascertain the places visited without adult accompaniment. This may be a possible solution for the lack of consensus about the operationalization of the level of independent mobility which limits the comparability between the results of this study and the results of other studies.

## Conclusion

A first conclusion of this study is that among 10–12 year old boys and girls, the parental perceptions of neighborhood environmental characteristics are associated with their level of active transport and total physical activity. This may be important for Belgian policy makers and urban planners to make well-considered decisions concerning built environmental redevelopments of existing Belgian neighborhoods and planning of new neighborhoods.

Secondly, in girls, the level of independent mobility plays a role in the association of the parental perceptions of neighborhood environmental characteristics with their level of active transport and their total physical activity level. When designing programs, focusing on neighborhood environmental characteristics, to promote active transport or physical activity, it should be kept in mind that the level of independence given to girls also needs to be considered to obtain behavior change. However, before the results of this study can be generalized, these results should be first confirmed in other studies.

## Competing interest

The authors declare that they have no competing interests.

## Authors’ contribution

FDM coordinated the data collection, assisted in the recruitment of the participants, conducted the statistical analyses and drafted the manuscript. GC, DVD and IDB participated in the interpretation of the data, revised the draft versions of the manuscript and provided critical comments during the process. All authors read and approved the final version of the manuscript.

## Pre-publication history

The pre-publication history for this paper can be accessed here:

http://www.biomedcentral.com/1471-2458/14/631/prepub
